# Egocentric Navigation Abilities Predict Episodic Memory Performance

**DOI:** 10.3389/fnhum.2020.574224

**Published:** 2020-11-27

**Authors:** Giorgia Committeri, Agustina Fragueiro, Maria Maddalena Campanile, Marco Lagatta, Ford Burles, Giuseppe Iaria, Carlo Sestieri, Annalisa Tosoni

**Affiliations:** ^1^Department of Neuroscience, Imaging and Clinical Sciences, University G. d'Annunzio, Chieti, Italy; ^2^Department of Psychology, University of Calgary, Calgary, AB, Canada

**Keywords:** egocentric navigation, path integration, episodic memory, semantic memory, medial temporal lobe

## Abstract

The medial temporal lobe supports both navigation and declarative memory. On this basis, a theory of phylogenetic continuity has been proposed according to which episodic and semantic memories have evolved from egocentric (e.g., path integration) and allocentric (e.g., map-based) navigation in the physical world, respectively. Here, we explored the behavioral significance of this neurophysiological model by investigating the relationship between the performance of healthy individuals on a path integration and an episodic memory task. We investigated the path integration performance through a proprioceptive Triangle Completion Task and assessed episodic memory through a picture recognition task. We evaluated the specificity of the association between performance in these two tasks by including in the study design a verbal semantic memory task. We also controlled for the effect of attention and working memory and tested the robustness of the results by including alternative versions of the path integration and semantic memory tasks. We found a significant positive correlation between the performance on the path integration the episodic, but not semantic, memory tasks. This pattern of correlation was not explained by general cognitive abilities and persisted also when considering a visual path integration task and a non-verbal semantic memory task. Importantly, a cross-validation analysis showed that participants' egocentric navigation abilities reliably predicted episodic memory performance. Altogether, our findings support the hypothesis of a phylogenetic continuity between egocentric navigation and episodic memory and pave the way for future research on the potential causal role of egocentric navigation on multiple forms of episodic memory.

## Introduction

Spatial navigation is a fundamental skill of all animal species that allows exploration, wayfinding, and homing (Montello, 2005). This ability depends on the integrity of a dedicated medial parieto-temporal neural network, which connects key structures of the medial temporal lobe (MTL), including the hippocampus, with more posterior brain regions such as the retrosplenial and the posterior cingulate cortex (Kravitz et al., 2011). In humans, different navigational strategies and processes have been described (Iaria et al., 2003; Igloi et al., 2009; Boccia et al., 2014), relying on either egocentric self-centered information or allocentric map-like information. Further studies have documented a large inter-individual variability in navigational skills (Hegarty et al., 2018), which is associated with differences in hippocampal gray matter volume (Wegman et al., 2014) and measures of resting-state functional connectivity (Arnold et al., 2014a; Sulpizio et al., 2016).

The MTL circuitry is also critical for the expression of declarative memory, which is similarly characterized by large inter-individual variability, as demonstrated, for instance, by individuals with extraordinary memory abilities (LePort et al., 2012; Dresler et al., 2017). Notably, the use of spatial learning strategies engaging the MTL is a main factor that contributes to superior memory (Maguire et al., 2003). Declarative memory has been classically divided into an episodic component, defined by a spatio-temporal connotation and a first-person perspective (i.e., self-based), and a semantic component, which refers to general knowledge independent of the temporal context and the individual's experience (i.e., map-based) (Tulving, 1983). While the MTL is crucial for episodic memory (Milner, 2005), its role in semantic memory has been debated (e.g., Kinsbourne and Wood, 1975; Squire and Zola, 1996). However, recent experimental evidence suggests that it makes necessary contributions to both types of declarative memory (reviewed by Duff et al., 2020).

For many years, navigation and memory have been investigated in independent lines of research, with major contributions from animal neurophysiological studies on spatial navigational mechanisms on one side, and neuropsychological assessment of amnesic patients on the other (for reviews see Eichenbaum, 2001, and Ekstrom and Isham, 2017). This separation resulted in a division among theories of hippocampal function, each one emphasizing its respective functional domain. However, based on the neuro-functional correspondence and organizational similarity at the level of the MTL, recent works have proposed a unified framework for navigational and memory functions. These accounts highlight the role of the hippocampus in the encoding of a wide variety of information, from present spatio-temporal contexts to events in abstract space (Schiller et al., 2015), and share the idea that spatial mechanisms can be applied to non-spatial domains, providing the building blocks for core elements of human thought and cognitive spaces (Epstein et al., 2017; Bellmund et al., 2018). Among these models, Buzsáki and Moser (2013) proposed a phylogenetic continuity between the neural mechanisms underlying navigation in the physical and mental (i.e., memory) space. In this view (see also Moser et al., 2015), episodic memory evolved from egocentric self-based navigation, such as path integration (i.e., the continuous updating of position and orientation during whole-body movement in space), whereas semantic memory evolved from allocentric map-based navigation.

Here, we tested whether traces of this phylogenetic continuity can be also observed in human behavior. Specifically, under the rationale that self-based and temporally-defined information processing in episodic memory closely resembles the way in which location sequences are linked together by a path integrator during egocentric navigation, we predicted that performance on an egocentric navigation task would be correlated with performance on an episodic memory task, but not with a semantic memory task. To test this hypothesis, we asked a sample of 60 participants to perform a proprioceptive path integration and a picture recognition task and administered a verbal semantic memory task as a control. We used robust partial correlation analyses to examine the degree of association between the task scores and used a leave-one-out cross-validation analysis to examine the predictive power of egocentric navigation on episodic memory performance. In a subset sample of participants (*N* = 30), we controlled for possible confounding effects of attention and working memory and we further tested for the degree of generalization across different types of path integration (by including a visual version of the task) and for the specificity of the egocentric relationship (by including a visual semantic memory task).

## Materials and Methods

### Main Experiment

#### Participants

The study was conducted on a sample of 60 healthy volunteers (mean age = 24.5 ± 3, 37 females) with reported normal or corrected-to-normal vision and no history of vestibular disease. Fifty-eight participants were right-handed while two were left-handed. Study participants were recruited from students enrolled in different courses of the University G. d'Annunzio of Chieti-Pescara (13–18 years of formal education). Participants were naïve as to the purpose of the experiment and were enrolled in the study after providing informed consent. The study was conducted in accordance with the ethical standards of the 1964 Declaration of Helsinki and was approved by the University Ethics Committee (prot. #1932 approved on July 11, 2019).

#### Cognitive Tasks

##### Proprioceptive triangle completion task (pTCT)

**Figure 1:**
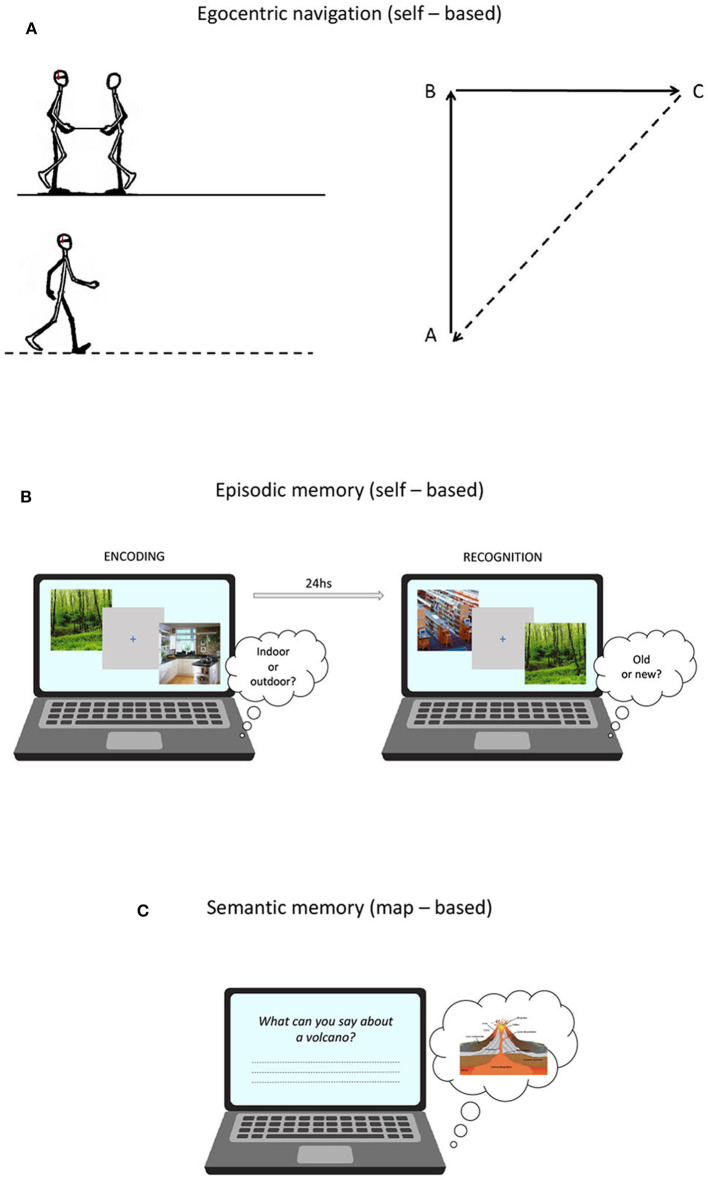
**(A)** Proprioceptive Triangle Completion Task (pTCT) used to asses egocentric navigation; **(B)** Picture Recognition Task (PRT) used to asses episodic memory; **(C)** General Knowledge of the World questionnaire (GKW) used to asses verbal semantic memory.

Egocentric navigation abilities were assessed using a task of proprioceptive path integration, which is defined as the capacity of monitoring self-motion to keep track of changes in orientation and position (Wolbers et al., 2007). The task was adapted from Wiener et al. (2011) and was performed in an ecological setting. Participants, blindfolded and wearing headphones emitting white noise, were led by the experimenter along the two sides of a triangle before autonomously returning to the starting position. The experimenter guided the participant along the path holding one end of a stick, whose other end was held by participants with both hands. At the beginning of each trial, the start position was indicated by the experimenter by means of two taps on the participant's shoulder. The stick was tugged lightly twice to indicate to start walking and tilted upward and rotated to indicate a change of direction. At the end of each path, a slowdown of the bar prompted the participant to orient toward and return to the starting position by following a direct way (Figure 1A).

Participants performed 16 triangulations differing for: (i) length of the sides of the triangles (first: 390 or 780 cm; second: from 276 to 872 cm), (ii) rotation directions (left or right), (iii) first turning angle (from 45° to 161°), (iv) homing angle (from 90° to 153°), and (v) homing distance (from 276 to 616 cm). The performance was recorded in terms of proportional distance error from the homing position, weighted by the homing distance. The details of each triangulation are reported in Supplementary Table 1.

##### Picture recognition task (PRT)

A measure of episodic memory performance was obtained using a picture recognition task (PRT) developed by Sestieri et al. (2014). The task is composed of two sessions (encoding and retrieval) separated by ~24 h (Figure 1B) and involves the manipulation of evidence (i.e., difficulty) for old and new responses (see Sestieri et al., 2014 for more details). At encoding, subjects made indoor/outdoor decisions on visually presented images depicting scenes from different categories. Four images from each of 60 categories were presented at varying frequency (1x, 3x, 5x) to modulate encoding strength and thus the evidence for old responses in the retrieval session. Each trial started with a 500 ms warning red fixation cross on a gray background, followed by the presentation of the image for 1 s and then by a 1 s blue fixation cross. Subjects had 2 s from image onset to provide a button press response. The order of trials (*N* = 600) was randomized in 15 experimental blocks. In the retrieval session, participants made item recognition judgments. Lures differed in their level of categorical and perceptual similarity with previously encoded pictures, resulting in increasing evidence for new responses. Each trial started with a 500 ms warning red cross on a gray background, followed by the presentation of the image for 1 s and then by a 2 s blue fixation cross. Subjects had 3 s from image onset to provide a button press. The order of trials (*N* = 360) was randomized in 12 experimental blocks. For data analyses, a d prime score (d') was calculated by assigning the label “hits” and “false alarms” to correctly identified old items and incorrectly identified new items, respectively. While the paradigm provides a measure of performance separately for the three levels of evidence/difficulty, only the global d prime (i.e., collapsed across evidence levels) was used as the dependent variable in the current work.

##### General knowledge of the world (GKW)

The standardized questionnaire of General Knowledge of the World (GKW) (Mariani et al., 2002) provided a measure of semantic memory performance. The test comprises 168 questions exploring 14 domains (12 questions each) of incidental and encyclopedic knowledge. Each answer is rated from 0 to 2, according to scoring procedures based on the level of specificity. The total score was corrected for age, education and gender (Mariani et al., 2002) (Figure 1C).

#### Apparatus and Procedure

The pTCT was collected in a 864 × 483 cm (*N* = 30) or a 1200 × 1700 cm (*N* = 30) empty room. The GKW and the PRT were, respectively, completed on Microsoft Word (Office 365 ProPlus) and E-Prime (2.0.10.356). The GKW and the PRT were performed on a 15″ (1024 × 768 pixels) laptop at a distance of ~60 cm from the screen. Participants completed the tasks in two consecutive days. The first day included the encoding phase of the PRT and the pTCT for a total session duration of ~2 h while the second day included the retrieval phase of the PRT (~50 min) and the GKW (variable duration: 1–4 h).

#### Statistical Analysis

A normality test was conducted for all the dependent variables (i.e., pTCT, PRT, GKW) using the Kolmogórov-Smirnov test on IBM SPSS Statistics 25.

Inferential statistics were conducted using Pearson Product-Moment Correlations tests with the associated *p*-values used to reject vs. accept the null hypothesis of an absence of correlation. Specifically, we examined the correlation between the accuracy scores associated with egocentric navigation (i.e., pTCT) and episodic memory (i.e., PRT) using the robust correlation analysis method implemented in Matlab R2020a (Pernet et al., 2013). Correlation values were obtained through skipped correlation analyses, which estimate the robust center of the data and associated Pearson correlation values after the automatic removal of bivariate outliers (Rousseeuw, 1984; Rousseeuw and Van Driessen, 1999; Verboten and Hubert, 2005).

A leave-one-out cross-validation analysis was also conducted using Matlab (R2020a) to test the predictive power of the pTCT over the PRT task. The analysis pipeline was based on a leave-one-subject-out cross-validation scheme in which each individual score in the pTCT was used to predict the corresponding episodic memory (i.e., PRT) score on the basis of a regression curve estimated from the remaining subjects. A Pearson correlation analysis was conducted between the observed and the predicted episodic memory scores. In order to account for the non-independence of the leave-one-out folds, we conducted a permutation test by randomly shuffling the pTCT scores 1,000 times and rerunning the prediction pipeline to create a null distribution of *r* values. The resulting *p*-value was based on the proportion of null distribution *r* values higher or equal to the corresponding empirical correlation value (see Shen et al., 2017; Beaty et al., 2018).

In addition, we examined the specificity of the relationship between egocentric navigation and episodic memory by conducting a series of analyses that included semantic memory: a robust correlation analysis between egocentric navigation (i.e., pTCT) and semantic memory (i.e., GKW) and two partial correlation analyses. In particular, we controlled for the effect of the semantic memory over the relationship between egocentric navigation and episodic memory by using the GKW score as a covariate in the Pearson correlation test between the pTCT and the PRT scores (IBM SPSS Statistics 25). Similarly, we controlled for the effect of the episodic memory over the relationship between egocentric navigation and semantic memory by conducting a partial correlation analysis between the pTCT and the GKW score with the PRT score as a covariate.

Partial correlations were conducted after the exclusion of the bivariate outliers identified by the original robust correlation analyses between the main two variables (i.e., pTCT&PRT, pTCT&GKW). Correlation values were finally compared using a dedicated analysis toolbox (see Hittner et al., 2003; Diedenhofen and Musch, 2015 for details).

### Additional Measures on a Subgroup of Subjects

#### Participants

We collected additional behavioral measures in a subset of the original sample (*N* = 30, mean age = 23.9 ± 2.26, 19 females) to examine whether the obtained results could be explained by individual differences in attention or working memory skills, and whether the results generalized to other perceptual domains.

##### Visual triangle completion task (vTCT)

A virtual reality version of the path integration task provided a measure of visual egocentric navigation (vTCT; Arnold et al., 2014b). Participants passively viewed themselves traveling along a virtual path in an empty desert-like environment from a first-person perspective on a computer screen. Each path consisted of two linear translations with one turn in-between. At the end of the guided path, participants used the keyboard to turn and then move forward to the estimated starting location. To respond accurately, participants are required to integrate the optic flow information provided during the passive movement phases to track their displacement and orientation relative to the starting point of each trial. A total of 16 trials were presented in randomized order with a total duration of ~10 min. Performance was defined in terms of absolute distance error, calculated as the difference between the ideal vs. the actual translation magnitude weighted by the correct return line distance in each trial (see Supplementary Figure 1 and Supplementary Table 2 for details about the virtual environment and individual trials, respectively).

##### Pyramids and palm trees test (PPT)

The Italian version (Gamboz et al., 2009) of the *Pyramids and Palm Trees Test* (PPT, Howard and Patterson, 1992) provided a measure of non-verbal semantic memory. The test is based on access to detailed semantic knowledge to identify analogies between pictures. We used a digitalized version of the traditional procedure in which a picture was presented on the top of the screen flanked by two other pictures on the bottom. Participants performed 52 trial, with a total duration of ~10 min, in which they indicated, using a computer keyboard, which of the two pictures at the bottom was semantically related to the one at the top. Accuracy was defined in terms of the proportional accuracy score.

##### Trail making test (TMT)

Performance on the Trial Making Test (TMT), including measures of visual search, scanning, speed of information processing and executive functions (Mondini et al., 2011), served as a control measure for the possible confounding effect of visual attention. Participants completed each part as quickly and accurately as possible. In the TMT A, they had to connect 25 encircled numbers on a sheet without removing the pen from the paper. In the TMT B, they alternated between numbers and letters (e.g., 1, A, 2, B…). The dependent variable reflects the time required to complete the TMT B. Task duration was ~5 min.

##### Memory with interference (MWI)

The possible confounding effect of working memory was controlled by measuring the ability to recall trigrams (meaningless three-consonant syllables, e.g., PMT) while counting forward by two from a random number for 10 or 30 s (in the first and second part of the test, respectively), to prevent rehearsal (Mondini et al., 2011). The dependent variable reflects the number of letters and trigrams (letter + presentation order) correctly recalled in both parts. The task duration was ~8 min.

#### Apparatus and Procedure

The tasks were administered using the same apparatus described above. Participants completed the tasks on two consecutive days. The PPT, TMT, MWI tasks were performed during the same day as the pTCT and the encoding phase of the PRT (first day), whereas the vTCT was performed the same day as the recognition phase of the PRT and the GKW (second day).

#### Statistical analysis

As in the previous set of analyses conducted on the whole sample, a normality test was conducted for all the dependent variables (pTCT, PRT, GKW, vTCT, PPT, TMT, MWI). For non-normally distributed variables, data were normalized using a log scaling function before conducting Pearson correlation tests. When variables remained non-normally distributed after the normalization procedure, a Spearman correlation test was additionally conducted.

We measured the correlation between accuracy of egocentric navigation (i.e., pTCT) and episodic memory (i.e., PRT) using a partial correlation analysis in which attention (i.e., TMT B) and working memory (i.e., MWI) scores were included as covariates. Bivariate outliers, previously identified in a robust correlation analysis between the two main variables (i.e., pTCT, PRT), were excluded from the analysis. We also conducted the same correlation analyses for the visual egocentric navigation scores (i.e., vTCT) and the non-verbal semantic memory task (i.e., PPT).

##### Correction for multiple comparisons

The results of all the correlation tests between egocentric navigation and episodic memory conducted in the present study, including partial correlations (a total of 15 tests), were corrected for false discovery rate (Benjamini and Hochberg, 1995) using the *fdr_bh* script running on Matlab (R2020a).

## Results

**Figure 2:**
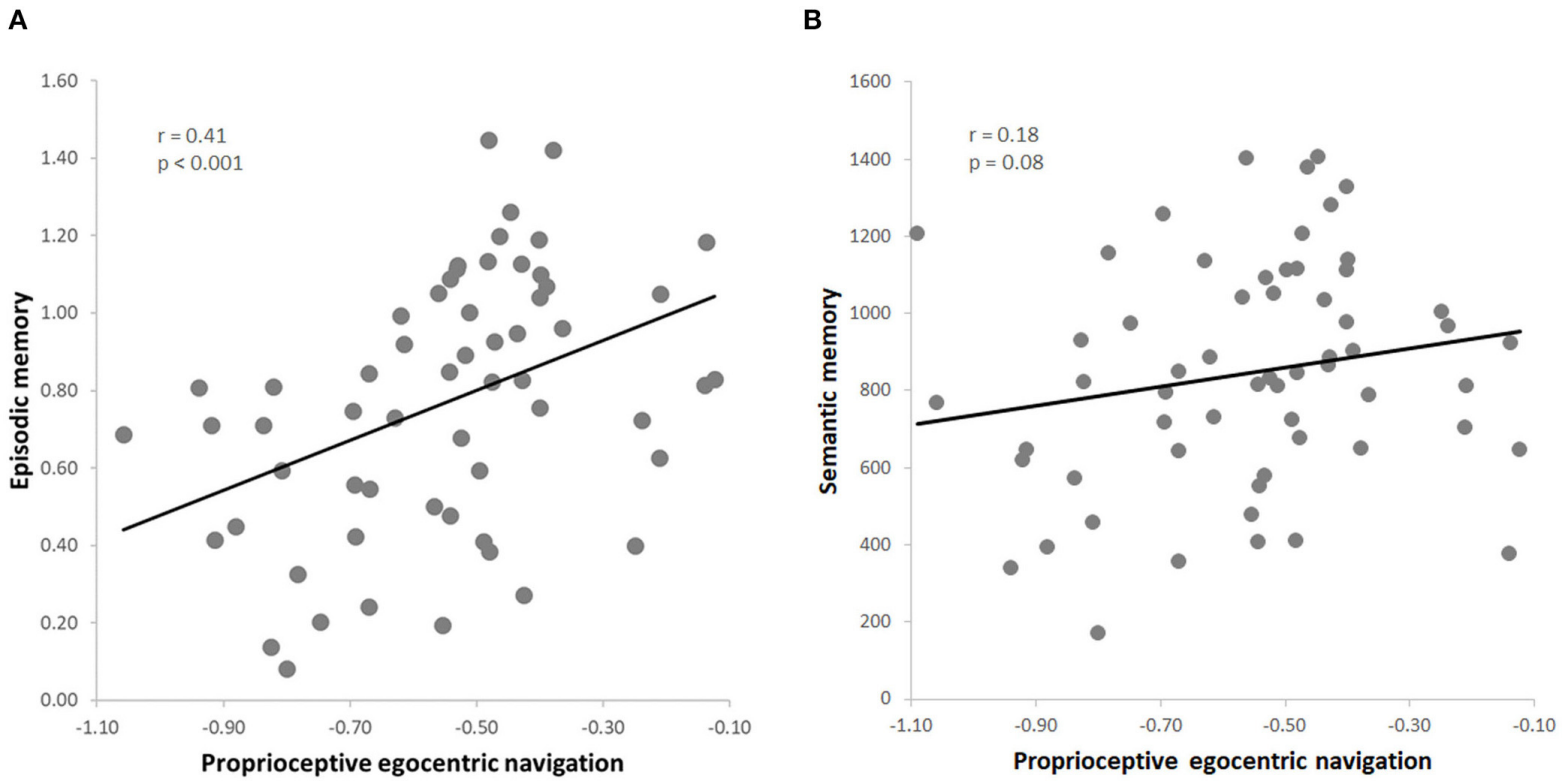
**(A)** Skipped correlation between proprioceptive egocentric navigation (pTCT) and episodic memory (PRT); **(B)** skipped correlation between proprioceptive egocentric navigation (pTCT) and verbal semantic memory (GKW).

The dependent variables obtained in the whole group of 60 subjects (pTCT, PRT, GKW) were normally distributed (all *p* > 0.05). Consistent with our predictions, the results indicated a positive correlation between egocentric navigation and episodic memory (*r* = 0.41, *p* < 0.001, FDR = 0.01) (Figure 2A). The leave-one-out cross-validation analysis additionally revealed that the episodic memory performance was reliably predicted by the egocentric navigation behavioral scores (*p* < 0.001).

Importantly, the analyses also indicated that this relationship was specific for episodic memory, as no significant correlation was observed between egocentric navigation and semantic memory (*r* = 0.18, *p* = 0.08) (Figure 2B). Since a positive correlation was found between the two declarative memory tasks (i.e., PRT, GKW) (*r* = 0.29, *p* = 0.01), we conducted a series of partial correlation analysis to test the relationship between egocentric navigation and each type of memory (i.e., PRT, GKW) while excluding the possible effect of the other memory (i.e., GKW, PRT). The correlation between egocentric navigation and episodic memory scores remained significant after the inclusion of the semantic memory scores as a covariate (*r* = 0.37, *p* = 0.004, FDR = 0.02). Also, the correlation between egocentric navigation and semantic memory remained not significant when controlling for episodic memory (*r* = 0.09, *p* = 0.51). Moreover, the first partial correlation was significantly stronger than the second one (*z* = 1.85, *p* = 0.03) (Hittner et al., 2003).

The normality test indicated that also the main dependent variables (pTCT, PRT, GKW, vTCT) collected in the subset of 30 participants that underwent the additional testing procedure were normally distributed (all *p* > 0.05). Out of the additional scores, however, the control measures of attention (i.e., TMT B) and working memory (i.e., MWI) and the non-verbal measure of semantic memory (i.e., PPT) did not show a normal distribution (TMT B, *p* = 0.001; MWI, *p* = 0.001; PPT, *p* = 0.002). As indicated in the Methods section, a log scaling function was applied, which resulted in a normalization of both the attention (*p* = 0.08) and the non-verbal semantic memory (*p* = 0.18) scores but not of the working memory scores (*p* = 0.002). A Spearman correlation test was conducted in addition to the Pearson correlation analysis to assess correlations involving this non-normal variable (MWI).

As expected, we observed a significant correlation between proprioceptive egocentric navigation scores and episodic memory performance when limiting the test on this subgroup of participants (*r* = 0.41, *p* = 0.01, FDR = 0.02). The correlation between proprioceptive egocentric navigation and episodic memory remained significant even when controlling for attention (*r* = 0.39, *p* = 0.04, FDR = 0.04) and working memory (Pearson's *r* = 0.37, *p* = 0.04, FDR = 0.04; Spearman's rho = 0.52, *p* = 0.005, FDR = 0.02) scores and the result was also replicated when including both covariates in the correlation analysis (Pearson's *r* = 0.38, *p* = 0.05, FDR = 0.05; Spearman's rho = 0.47, *p* = 0.01, FDR = 0.02).

**Figure 3:**
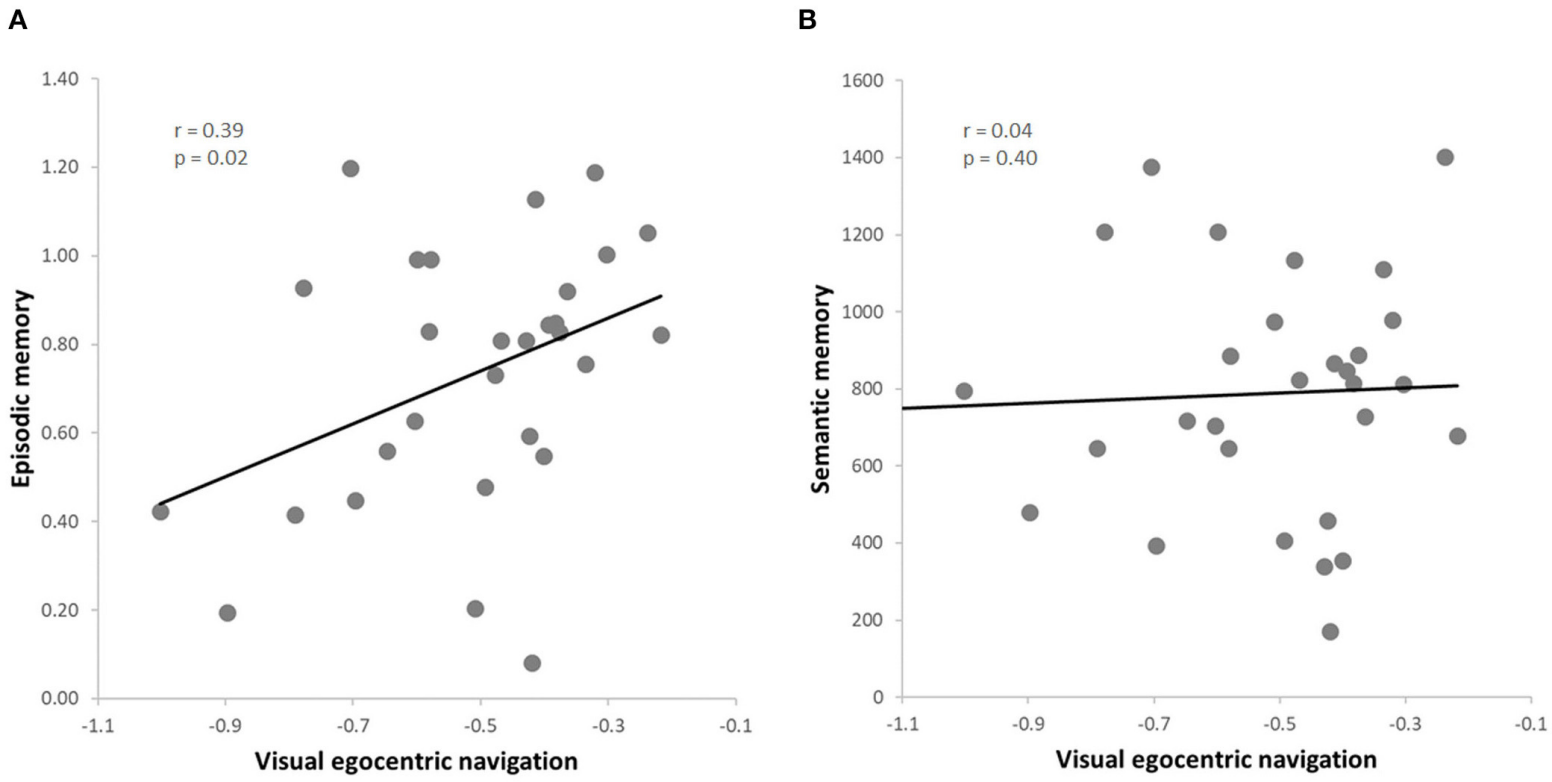
**(A)** Skipped correlation between visual egocentric navigation (vTCT) and episodic memory (PRT); **(B)** Skipped correlation between visual egocentric navigation (vTCT) and verbal semantic memory (GKW).

The data from this subgroup further allowed to extend the results to the visual domain of egocentric navigation (i.e., vTCT). Once again, we observed a positive correlation between the visual egocentric navigation and the episodic memory scores (*r* = 0.39, *p* = 0.02, FDR = 0.04) (Figure 3A), also when controlling for the semantic memory (i.e., GKW) performance (*r* = 0.46, *p* = 0.01, FDR = 0.02). Moreover, as expected on the basis of the previous analysis on the whole sample, no significant correlation was found between the visual egocentric navigation and the semantic memory scores (*r* = 0.04, *p* = 0.40) (Figure 3B), also when controlling for episodic memory performance (*r* = −0.23, *p* = 0.22). As before, we observed a significant difference between the two correlation values (vTCT & PRT > vTCT & GKW; z = 4.3877, *p* < 0.001) (Hittner et al., 2003). The correlation analysis between the visual egocentric navigation and the episodic memory scores indicated a significant correlation also when controlling for attention (*r* = 0.39, *p* = 0.04, FDR = 0.04) and working memory (*r* = 0.43, *p* = 0.02, FDR = 0.04; rho = 0.42, *p* = 0.03, FDR = 0.04) and the result was also replicated when including both covariates in the analysis (*r* = 0.42, *p* = 0.03, FDR = 0.04; rho = 0.39, *p* = 0.04, FDR = 0.04).

Finally, with reference to the non-verbal semantic memory control task (i.e., PPT), we found no significant correlation with either the proprioceptive (i.e., pTCT) (*r* = 0.08, *p* = 0.70) or the visual (i.e., vTCT) (*r* = 0.05, *p* = 0.81) egocentric navigation while including the episodic memory scores as a covariate.

## Discussion

The medial temporal lobe is long known to support both navigation and declarative memory. Following considerations on the neural mechanisms supporting these two crucial functions, Buzsáki and Moser (2013) developed a model of phylogenetic continuity between specific mechanisms of navigation in the physical world (i.e., self-based vs. map-based navigation) and different types of declarative memory (episodic vs. semantic, respectively). Here, we tested whether traces of such evolutionary bond are still detectable in human behavior by designing a behavioral study assessing human abilities in different navigational and memory tasks. Notably, since the model predictions assume that each form of high-level representation (i.e., map-based allocentric navigation, semantic memory) derives from its corresponding low-level representation (self-based egocentric navigation, episodic memory), we specifically focused on the relationship between egocentric navigation and episodic memory performance.

To this aim, analyses were conducted to examine the degree to which performance during an egocentric navigation task (i.e., path integration) is associated and predicts performance during an episodic memory task (i.e., picture recognition). Importantly, we investigated the specificity of such a relationship by including measures of semantic memory in the study design. Consistent with our hypothesis, we found that participants who exhibited better performance in the path integration task also exhibited a more accurate remembering of previously encoded items. This finding was supported by both a robust correlation and a predictive cross-validation analysis. Notably, the relationship was significant regardless of whether path integration was based on proprioceptive or visual inputs. In contrast, no significant correlation was found between the path integration and either the verbal or non-verbal semantic memory performance. The specificity of the positive relationship between egocentric navigation and episodic memory was further supported by the statistical independence of the observed correlation from basic attentional or working memory functions.

We propose that the observed behavioral relationship between self-based navigation and episodic memory functions rely on a substantial overlap between the underlying neuronal computations within the medial temporal lobe. In particular, by coding both locations in space and moments in time, the hippocampus can be thought of as a core structure for the encoding, representation and retrieval of spatio-temporal relations (Eichenbaum, 2014; Epstein et al., 2017; Moser et al., 2017; Ekstrom and Ranganath, 2018). More compelling for the egocentric path integration, it is widely known that enthorinal physiological mechanisms (i.e., persistent spiking and membrane potential oscillations) are able to generate sequential or ramping activity patterns that carry time-related information. This neural pattern might critically support the encoding and retrieval of trajectories through space and time (Hasselmo and Brandon, 2008; Issa et al., 2020).

Alternatively, based on the work by Arnold et al. (2014b) on the neural correlates of visual path integration, it might be suggested that performance in the two tasks is supported by common computations orchestrated in a wider brain network, including posterior parietal and prefrontal regions associated with attentional and spatial working memory functions. However, the independence of the observed relationship from basic attentional and working memory functions is not consistent with such a hypothesis.

We acknowledge that the current data, and in particular the findings of a non-significant correlation between navigation and semantic memory, do not allow to exclude the possibility that other navigational mechanisms are specifically linked with the organization of semantics and abstract thought. As noted earlier, the continuous population code of place and grid cells in the hippocampus-entorhinal system appears to contain a suitable organization for the mapping of several dimensions of the cognitive space, and thus to retain a general role in several aspects of human cognition (e.g. Bellmund et al., 2018). Accordingly, a recent neuroimaging study has shown that the same brain regions and neural codes supporting spatial navigation are recruited when humans use language to organize new semantic representations and that neural data could be reliably used to reconstruct the between-concepts relationship in memory (Viganò and Piazza, 2020). However, rather than investigating a learning process of new semantic information, here we emphasized the contribution of crystallized semantic memory. On this basis, we speculate that the ability to transform new episodic experience into long-term semantic memory might be correlated with navigational abilities associated with egocentric-to-allocentric transformation, such as those activated during the acquisition of a survey representation from route-based learning. Old semantic knowledge might instead heavily rely on allocentric, map-based navigational mechanisms, as suggested by the original proposal of Buzsáki and Moser (2013), but dedicated studies are needed to address this hypothesis.

Of note, since the present study was the first explicit behavioral investigation of the egocentric navigation-episodic memory relationship, we opted for a basic episodic memory task (i.e., item recognition), which did not require the construction of a time sequence or a mental-time navigation process. Although we believe that our strategy was critical to control potential confounds associated with the use of spatial strategies across tasks, it would be also interesting to investigate the same relationship using tasks of temporal order memory or mental time travel. Moreover, it would be also worth testing potential clinical applications of the observed predictive relation between navigational and memory functions, by training participants on egocentric navigation and assessing eventual positive effects (i.e., empowerment) on episodic memory performance. For example, individuals with episodic memory deficits, such as patients with dementia and schizophrenia (Tromp et al., 2015; Kanchanatawan et al., 2018; Das et al., 2019), might benefit from a training of their primal navigational system as an indirect way of boosting their episodic memory system. In particular, a recent single-case report on a patient suffering from topographical disorientation has demonstrated the positive effect on episodic memory of an imagery-based navigational training (Boccia et al., 2019).

In conclusion, we offer compelling behavioral evidence about a specific and predictive relationship between egocentric, self-based navigation abilities and episodic memory performance, providing support to the hypothesis that the ability to navigate in mental space evolved from recycling mechanisms developed for navigating in physical space.

## Data Availability Statement

The raw data supporting the conclusions of this article will be made available by the authors, without undue reservation.

## Ethics Statement

The studies involving human participants were reviewed and approved the Ethics Committee for Biomedical Research of the provinces of Chieti and Pescara, and of the University of studies G. d'Annunzio of Chieti and Pescara (prot. #1932 approved on July 11 2019). The patients/participants provided their written informed consent to participate in this study.

## Author Contributions

GC, AF, AT, and CS designed the study. AF, MC, ML, and FB collected and analyzed the data. GC and AF drafted the manuscript. GC, AF, GI, CS, and AT revised the manuscript. All authors contributed to the article and approved the submitted version.

## Conflict of Interest

The authors declare that the research was conducted in the absence of any commercial or financial relationships that could be construed as a potential conflict of interest.
