# Supplementary material for: Egocentric Navigation Abilities Predict Episodic Memory Performance
Source: Front Hum Neurosci. 2020 Nov 27;14:574224. doi: 10.3389/fnhum.2020.574224 (PMC7729005; doi:10.3389/fnhum.2020.574224)
Supplement: Supplementary file 1 [file Table_1.DOCX]

**Supplementary materials**

**Table 1.** Proprioceptive Triangle Completion Task. Details of each triangulation with segments and distance expressed in centimeters and the rotation angles in degrees.

| **TRIAL** | **1st segment** | **1st turn angle** | **2nd segment** | **Return turn angle** | **Homing distance** |
| --- | --- | --- | --- | --- | --- |
| A | 390 | 135 | 551 | 135 | 390 |
| B | 390 | 135 | 275 | 90 | 275 |
| C | 390 | 90 | 390 | 135 | 551 |
| D | 390 | 45 | 275 | 153 | 616 |
| E | 780 | 153 | 872 | 116 | 390 |
| F | 780 | 161 | 616 | 63 | 275 |
| G | 780 | 135 | 551 | 90 | 551 |
| H | 780 | 135 | 275 | 63 | 616 |
| I | 390 | -135 | 551 | -135 | 390 |
| J | 390 | -135 | 275 | -90 | 275 |
| K | 390 | -90 | 390 | -135 | 551 |
| L | 390 | -45 | 275 | -153 | 616 |
| M | 780 | -153 | 872 | -116 | 390 |
| N | 780 | -161 | 616 | -63 | 275 |
| O | 780 | -135 | 551 | -90 | 551 |
| P | 780 | -135 | 275 | -63 | 616 |

**Table 2.** Visual Triangle Completion Task. Details of each trial with the translations and distance expressed in milli-virtual-units and the rotation angles in degrees. For more information: <https://fordburles.com/path-integration-task.html>

| **TRIAL** | **1st translation** | **1st turn angle** | **2nd translation** | **Return turn angle** | **Homing distance** |
| --- | --- | --- | --- | --- | --- |
| A | 4000 | -135 | 5657 | -135 | 4000 |
| B | 4000 | -90 | 4000 | -135 | 5657 |
| C | 4000 | -135 | 2828 | -90 | 2828 |
| D | 4000 | -45 | 2828 | -153 | 6325 |
| E | 4000 | 135 | 5657 | 135 | 4000 |
| F | 4000 | 90 | 4000 | 135 | 5657 |
| G | 4000 | 135 | 2828 | 90 | 2828 |
| H | 4000 | 45 | 2828 | 153 | 6325 |
| I | 8000 | -153 | 8944 | -117 | 4000 |
| J | 8000 | -135 | 5657 | -90 | 5657 |
| K | 8000 | -162 | 6325 | -63 | 2828 |
| L | 8000 | -135 | 2828 | -63 | 6325 |
| M | 8000 | 153 | 117 | 117 | 4000 |
| N | 8000 | 135 | 5657 | 90 | 5657 |
| O | 8000 | 162 | 6325 | 63 | 2828 |
| P | 8000 | 135 | 2828 | 63 | 6325 |

**Figure 1.** A sample screenshot of the virtual environment used in the Visual Triangle Completion Task. The environment does not contain any salient landmarks or distal directional cues.

**
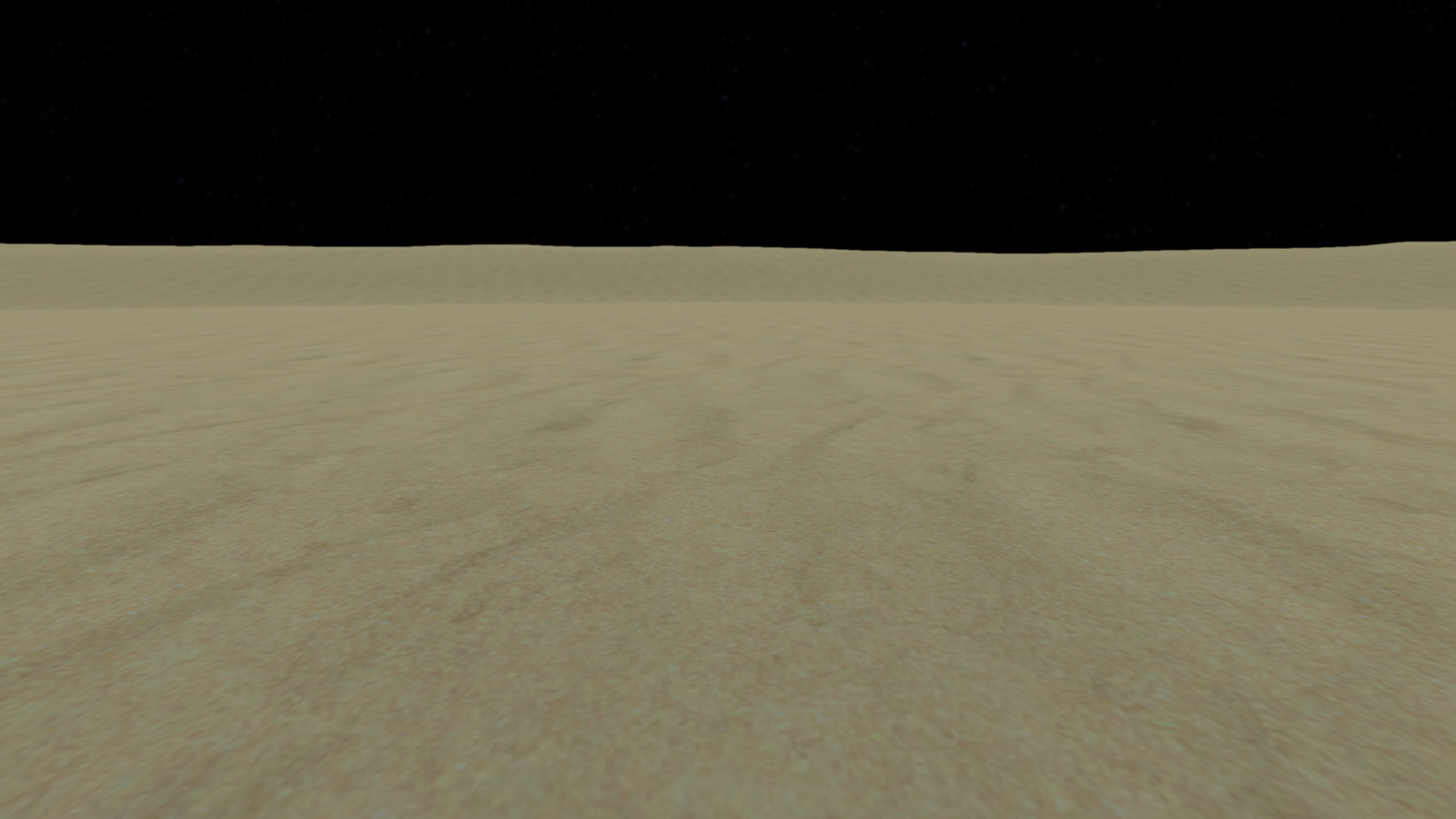
**
